# Effect-less? Event-files are not terminated by distal action effects

**DOI:** 10.3758/s13414-023-02754-w

**Published:** 2023-07-07

**Authors:** Christian Frings, Silvia Selimi, Paula Soballa, Daniel H. Weissman

**Affiliations:** 1 Faculty of Behavioral Sciences, Department of Psychology, University of Trier, Universitätsring 15, D-54296 Trier, Germany; 2 Department of Psychology, University of Michigan, 1004 East Hall 530 Church Street, Ann Arbor, MI 48109-1043 USA

**Keywords:** Perception and action, Action effects, Event-file termination

## Abstract

Event-files that bind features of stimuli, responses, and action effects figure prominently in contemporary views of action control. When a previous feature repeats, a previous event-file is retrieved and can influence current performance. It is unclear, however, what terminates an event-file. A tacit assumption is that registering the distal (e.g., visual or auditory) sensory consequences of an action (i.e., the “action effect”) terminates the event-file, thereby making it available for retrieval. We tested three different action-effect conditions (no distal action effect, visual action effect, or auditory action effect) in the same stimulus-response (S-R) binding task and observed no modulation of S-R binding effects. Instead, there were comparably large binding effects in all conditions. This suggests that proximal (e.g., somatosensory, proprioceptive) action effects terminate event-files independent of distal (e.g., visual, auditory) action effects or that the role event-file termination plays for S-R binding effects needs to be corrected. We conclude that current views of action control require further specification.

Event-files linking perception with action make important contributions to action control. Such files consist of temporary bindings – or associations – between features of stimuli, responses, and action effects, which are formed while planning and executing actions (Hommel, 2004). More specifically, according to the theory of event-coding (TEC) (Hommel, 1998, 2004; Hommel et al., 2001), event-files are short-lived episodes of feature compounds, which integrate object (Kahneman et al., 1992) with action files. Event-files are retrieved – and thereby influence action control – when one or more features from a previously bound event-file reappears during the current stimulus-processing episode. Current theorizing on action control is, therefore, concerned with the dynamic management of event-file binding and retrieval (e.g., the Binding and Retrieval in Action Control framework (BRAC); Frings et al., 2020).

One seldom-addressed issue concerns what terminates an event-file. As retrieving an event-file formed in trial n-1 influences responding in trial n (Hommel, 1998), researchers often assume that the event-file in trial n-1 was terminated. However, they do not specify what triggers event-file termination.^1^ They just tacitly assume that registering the sensory consequences of an action – also known as the “action effect” – somehow terminates the event-file. This assumption stems from the fact that both TEC and BRAC are rooted in ideomotor theory (Shin et al., 2010), which posits that humans plan actions by anticipating their effects. Consistent with ideomotor theory, both TEC and BRAC posit that, in a typical prime-probe action control task, the stimulus, response, and action effect in the prime trial (trial n-1) are bound into an event-file. The assumption that action effects terminate an event-file, however, is untested.

The need for such a test is indicated by mixed findings in the literature regarding whether event-file termination hinges on action effects. In line with this possibility, binding effects appear in prime-probe tasks wherein participants respond to stimuli that disappear as soon as a response is executed and, consequently, produce a distal – or environmental – action effect (i.e., erasure of the stimuli that participants responded to; (Frings & Moeller, 2010; Frings & Rothermund, 2017; Hommel, 1998). Moreover, in studies of Negative Priming (for an overview, Frings et al., 2015), which is also thought to rely on event-file formation and retrieval (Frings et al., 2020), display offset modulates performance (Frings & Wühr, 2007; Houghton & Tipper, 1994). In other studies, however, binding effects appear in tasks wherein participants respond well after the stimulus has disappeared, which does not produce a distal action effect (Grant et al., 2020; Weissman et al., 2020; Weissman et al., 2016). These findings suggest that event-file termination does not hinge completely on distal action effects. To our knowledge, however, no prior study has investigated whether the magnitude of binding effects varies with the presence or nature of distal action effects.

One possibility is that a combination of proximal and distal action effects leads to larger binding effects than proximal action effects on their own.^2^ Consistent with this view, some findings suggest that distal effects are often more salient or potent for action control than proximal effects. For instance, in studies of bimanual coordination it is the distal perceptual symmetry, not the proximal muscle or motor symmetry, that drives action (Mechsner et al., 2001). As another example, a recent review on motor performance and learning argues that it is mainly distal, external attentional focus – rather than proximal, internal focus – that drives motor performance (Chua et al., 2021). Finally, experiments on tool use suggest that anticipated visual effects in distal, external space play a prominent role in the selection, initiation, and actual execution of movements, while anticipated proximal effects are attenuated or ignored (Kunde et al., 2007; Massen & Prinz, 2007). These findings suggest that distal effects are more salient and, possibly, more impactful for action control than proximal effects. Therefore, they suggest that event-file termination should be stronger or more clear-cut when distal action effects are present than when they are absent, because the clearer an event-file is terminated the better it can be retrieved, ultimately resulting in larger binding effects.

Another possibility is that the magnitude of binding effects does not vary with the presence or absence of distal action effects. In line with this possibility, Pfister (2019) argued that every response elicits a proximal action effect, namely, the touch of the fingertip on the keyboard and the associated proprioceptive feedback. Thus, proximal action effects may terminate event-files on their own without an additional influence of distal (e.g., visual or auditory) action effects.

And finally, there are arguments that action effects do not per se terminate event-files but that event segmentation terminates an event-file (e.g., by prediction errors; Foerster et al., 2023; Foerster et al., 2022), and recent versions of the event-file concept seem to embrace this idea (e.g., Hommel, 2022). Related to this, accounts of action-effect monitoring (Wirth et al., 2018) argue that event-files may be kept open until registering anticipated action effects in the environment. We turn to these different views on event-file termination in the *Discussion* section.

Here we analyze the impact of distal action effects on S-R binding in the context of a typical sequential binding task. To this end, we manipulate whether stimuli are (i) erased at the response (visual action effect), (ii) erased before the response, which, however, triggers an auditory tone (auditory action effect), or (iii) erased before the response, which does not trigger a tone (no distal action effect). If distal action effects contribute to the termination of event-files, and if event-file termination eases retrieval, then binding effects should be greater in the visual and auditory action-effect conditions than in the no distal action-effect condition. Indeed, visual and auditory action effects should serve as additional, salient cues to terminate the event-file. To test this hypothesis, we measure binding as an interaction between stimulus relation (repeat, change) and response relation (repeat, change) across trial n-1 and trial n in a sequential prime-probe task (Hommel, 1998). The presence of this interaction implies that repeating a feature from trial n-1 in trial n retrieves the terminated event-file from trial n-1.

## Methods

### Participants

The sample size was calculated using previous S-R binding effects, which typically lead to medium effect sizes (*dz* around 0.5, e.g., Moeller et al., 2016). Given alpha = .05, one-tailed testing and a desired power of at least 1-*β* > 0.80, we aimed for a minimum of 27 participants (power analyses were conducted with GPower 3.1.9.7; Faul et al., 2007). To account for possible dropouts, 30 participants were tested. All participants (26 female, three left-handed, age range: 19–27 years, median age: 21.5 years) were students from the University of Trier who gained course credits for their participation and gave active informed consent prior to participation. One participant was excluded from analyses due to a high number of erroneous or missing responses (more than 62% of trials had to be discarded; binding effects could not be calculated, as there were no trials left in some sub-conditions). The experiment was conducted in line with the ethical guidelines of the German Psychology Association and the guidelines of the ethics committee of the University of Trier.

### Design

We used a 2 (stimulus relation: repetition vs. change) × 2 (response relation: repetition vs. change) × 3 (experimental condition: visual action effect, auditory action effect, no distal action effect) within-subjects design. Stimulus relation and response relation varied orthogonally on a trial-by-trial basis. Experimental condition varied across three separate blocks, the order of which was balanced across participants using a Latin square design. Binding effects were computed as the interaction of stimulus relation and response relation.

### Apparatus and stimuli

The experiment was programmed with PsychoPy (v2020.2.10; Peirce et al., 2019) and its online application PsychoJS, and hosted on Pavlovia.org. The cue stimuli were the signs “>” and “<”, which had a font size of 30 pixels. The target stimuli were the letters A, B, C, and D, which had a font size of 25 pixels. All stimuli were presented in white on a black background. As a tone effect, an “A” note was played as a sine tone (approx. 440 Hz). However, since the study was conducted online, we do not have knowledge of whether participants played the sounds via headphones or loudspeakers, and which volume they had set.

### Procedure

Instructions appeared on the screen. First, a test tone was played, and participants were advised to adjust the volume so they could hear the sound clearly. Second, participants were instructed to place their left and right index fingers on the F and J keys. Third, the tasks and prime-probe trial structure were explained.

Each prime-probe trial pair consisted of three events. First, there was a central fixation cross. Participants pressed the space bar to proceed. Second, one of the two cues appeared for 500 ms. The cue indicated whether to press the left key or the right key as quickly and accurately as possible when the trial n-1 letter appeared 500–1,000 ms later (varied in 50-ms steps). Third, 1,100 ms after the onset of the trial n-1 letter, the trial n letter appeared. Participants were told to press the left key if an A or a C appeared or the right key if a B or a D appeared.

There were four trial types. In stimulus-repeat trials (SR; 25% of all trials), the two letters were the same. In stimulus-change trials (SC; 75% of all trials) the two letters differed. In response-repeat trials (RR; 50% of all trials), the required response repeated between trial n-1 and trial n. In response-change trials (RC; 50% of all trials), the required response changed.

**Fig. 1:**
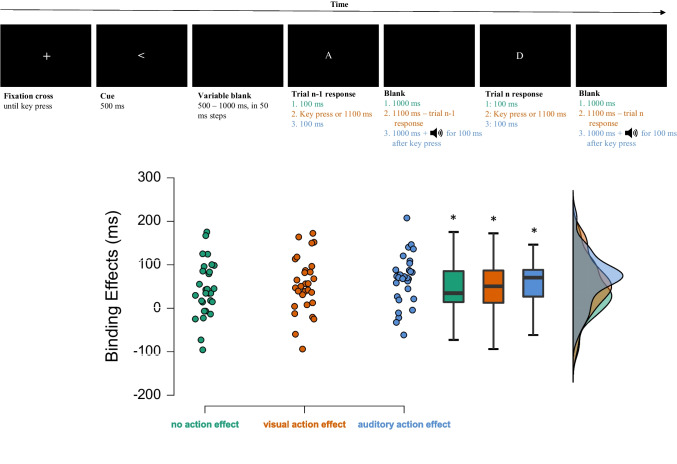
**Top:** Schematic trial sequence for the three conditions: (1) no distal action effect (green), (2) visual action effect (orange), and (3) auditory action effect (blue). Stimuli are not drawn to scale, see text for further explanations. **Bottom:** Binding effects in milliseconds as a function of experimental condition depicted at the level of individual effects (left), group-level box plots (middle), and frequency distributions (right). * *p* < .001

Responding produced different action effects in the three experimental conditions. In the “no distal action effect” condition, responding induced no distal action effect: each letter appeared for 100 ms and was followed by a 1,000-ms blank screen regardless of when the response occurred. In the “visual action effect” condition, each letter appeared for up to 1,100 ms but was immediately erased when a response occurred, leaving the screen blank until 1,100 ms post-stimulus onset. In the “auditory action-effect condition,” each letter appeared as in the “no distal action effect” condition, but a 100-ms tone played when participants responded (see Fig. 1).

Prior to the main experimental session, each participant worked through 32 practice trials from the “no distal action effect” condition. If a participant’s accuracy related to withholding the first response until the trial n-1 letter appeared and correctly executing both reactions was below 75%, then the participant had to repeat these 32 trials (14 participants). Otherwise, the participant continued to the main experiment, which consisted of three action-effect conditions. Each condition included a 32-trial practice block and a 96-trial test block (288 test trials total). Participants were given the opportunity to take a short break every 32 trials.

## Results

We analyzed performance in trial n. Trials with anticipated trial n-1 responses (4.9%) or missing responses in either trial n-1 or trial n (4.8%) were excluded. In the analysis of response time (RT), we considered only trials with correct responses in both trial n-1 and trial n. The mean error rates were 2.5% for the trial n-1 and 13.3% for the trial n (only counting trials with correct trial n-1 responses). Additionally, we excluded RTs of more than 1.5 interquartile ranges above the third quartile of the trial n response RT distribution of the participant (Tukey, 1977), and RTs shorter than 100 ms from the analysis. With these constraints, 19.8% of the trials were excluded from the RT analyses. For mean RTs and error rates (ERs), see Table 1.

**Table 1:** Mean response times (in ms) and mean error rates (in parentheses, %) for trial n responses, as a function of stimulus relation between trial n-1 and trial n, response relation, and experimental condition

	No distal action effect		Visual action effect		Auditory action effect	
	*RR*	*RC*	*RR*	*RC*	*RR*	*RC*
*SC*	467 (16.5)	444 (8.7)	577 (12.6)	551 (8.1)	465 (13.9)	442 (9.3)
*SR*	436 (6.9)	456 (23.2)	530 (5.0)	557 (20.8)	421 (6.8)	464 (22.1)

*RR* response repeat, *RC* response change, *SC* stimulus change, *SR* stimulus repeat

In a 2 (stimulus relation: repetition vs. change) × 2 (response relation: repetition vs. change) × 3 (experimental condition: no distal action effect, visual action effect, auditory action effect) analysis of variance (ANOVA) on trial n mean RT, the main effects of condition, *F*(2, 56) = 63.12, *p* < .001, *η*_*p*_^*2*^ = .69, and stimulus relation, *F*(1, 28) = 11.65, *p* = .002, *η*_*p*_^*2*^ = .29, were significant, while the main effect for response relation was not significant, *F*(1, 28) = 0.47, *p* = .500, *η*_*p*_^*2*^ = .02. Importantly, the interaction between stimulus relation and response relation was significant, *F*(1, 28) = 87.36, *p* < .001, *η*_*p*_^*2*^ = .76, indicating stimulus-response binding.

Critically, experimental condition did not modulate this interaction, *F*(2, 56) = 0.79, *p* = .457, *η*_*p*_^*2*^ = .03. Follow-up analyses revealed significant binding effects in the no distal action-effect condition, *t*(28) = 3.72, *p* <.001, *d* = 0.691, the visual action-effect condition, *t*(28) = 4.50, *p* <.001, *d* = 0.836, and the auditory action-effect condition, *t*(28) = 6.05, *p* <.001, *d* = 1.124 (see Fig. 1). A Bayesian ANOVA in JASP (JASP Team, 2022)^3^ revealed that the best model comprised all three main effects and the interaction between stimulus and response relation (BF_M_ = 108). Adding the three-way interaction made this model more than 100 times less likely (BF_01_ > 100 in several runs). These findings indicate a null modulatory effect of experimental condition on stimulus-response binding according to Bayesian conventions (Wagenmakers et al., 2018).

In the same analysis on trial n mean ER, the main effects of stimulus relation, *F*(1, 28) = 4.90, *p* = .035, *η*_*p*_^*2*^ = .15, and response relation, *F*(1, 28) = 6.91, *p* = .014, *η*_*p*_^*2*^ = .20, were significant, while the main effect of experimental condition did not reach significance, *F*(2, 56) = 1.85, *p* = .166, *η*_*p*_^*2*^ = .06. Again, the interaction between stimulus and response relation was significant, *F*(1, 28) = 69.29, *p* < .001 , *η*_*p*_^*2*^ = .71, but not further modulated by experimental condition, *F*(2, 56) = 0.52, *p* = .595, *η*_*p*_^*2*^ = .02. The same Bayesian ANOVA on error rates also revealed that the best model comprised main effects for stimulus and response relation as well as the interaction between stimulus and response relation (BF_M_ = 69). Adding the three-way interaction made this model more than 100 times less likely (BF_01_ > 150 in several runs).

## Discussion

The present findings indicate that binding effects do not vary with the presence or nature of distal action effects (see also Pfister et al., 2022, Appendix). Against the background of some current ideomotor theorizing (Frings et al., 2020; Hommel et al., 2001), this outcome is astonishing. Indeed, it suggests that event-file termination does not depend on the presence of distal action effects *or* – even more intriguing – that event-file termination does not play the role previous theorizing assumed. For instance, event-files may be terminated before, rather than after, an action is integrated with its distal effects, or event-files might not need to be terminated for retrieval at all. We discuss each of these possibilities next.

First, event-file termination may not hinge on distal action effects because relying on proximal action effects is more adaptive in real-world settings. Indeed, an action (e.g., lifting a finger in the dark, lightly touching a surface, etc.) may not always elicit a salient distal effect (e.g., a change in visual or auditory input). Or an action (e.g., typing) may elicit a salient distal effect that is not perceived (e.g., because a typist is listening to music over headphones while typing a report). Consequently, relying exclusively on proximal action effects to terminate event-files, as the present findings suggest, could be an adaptive “one-size-fits-all” approach for ensuring that recent experience exerts an influence on action control in real-world settings.

Event-file termination may also, or alternatively, not hinge on distal action effects because action effects terminate an event-file whenever the discrepancy between anticipated and future perceptual states reaches a value of zero (Kunde et al., 2017; Wirth et al., 2018). In the present study, subjects could accurately anticipate the future perceptual state they would experience after each response (whether there would be no distal action effect, a visual action effect, or an auditory action effect) because all of the action effects were fully predictable in our blocked design. Consequently, the discrepancy between anticipated and future states was zero as soon as a response occurred, regardless of the presence or absence of distal effects. Future work could be aimed at determining whether proximal action effects and/or minimizing the discrepancy between anticipated and future states can best explain the present findings by varying the predictability of the prime response.

Second, a more speculative explanation of our findings is that two event-files are created: a stimulus-response file and a response-effect file. In line with this view, although responses are bound to both (1) stimuli and (2) action effects, there are no bindings between stimuli and action effects (Moeller et al., 2019). The view can also explain our finding that action effects do not influence stimulus-response bindings. It does not, however, fit well with current views positing a single file (e.g. Frings et al., 2020). A related possibility is that the response terminates a single stimulus-response file. This view, however, does not explain how distal action effects enter event-files after such files are terminated, since the response is ultimately integrated with its effects (Dutzi & Hommel, 2009; Elsner & Hommel, 2001).

Finally, one might wonder whether event-file termination is in fact needed at all for event-files to be re-activated. In frameworks like TEC or BRAC, one can deduce that event-files are retrieved only after they are terminated. There is no clear-cut evidence, however, that event-files *must* be closed before retrieval can happen. Consequently, “still-open” event-files may be reactivated and updated when a probe display appears, as has been suggested in related fields (with somewhat other terminology of course, e.g., Treisman, 1996). In this regard, distal action effects can then just be seen as if they add more features that can be used to represent a response, and accordingly more opportunities for S-R bindings. The visual offset condition, for example, seems to link the response particularly closely to the stimulus because it can be represented as removing the stimulation. The added tone effect in the auditory condition may also been seen as adding a potential feature that could be used to represent the response, which can also enter bindings. However, these additional feature bindings do not relate to termination of the event-file. In our experiment, though, it did not make any difference whether these additional feature bindings were present or not.

In sum, we investigated a tacit assumption that is inherent in some approaches of contemporary action control research, which is that distal action effects are important for terminating event-files. Contrary to this assumption (Frings et al., 2020; Hommel et al., 2001), we found that a combination of proximal and distal action effects does not modulate S-R binding effects. These findings suggest that event-file termination (a) does not always depend on distal effects or (b) occurs before, rather than after, an action is integrated with its distal effects, or (c) is not needed for retrieval at all. More broadly, they raise fundamental questions about how event-files are formed and about how action effects are integrated with the response. The event-file concept is a milestone in perception-action integration, but our findings suggest that it requires further specification.

## Footnotes

1 Exceptions are studies focusing on more complex actions (Moeller & Frings, 2019) or variants of sequential action control tasks wherein the event-file in trial n-1 is not terminated because the response and action effects of trial n-1 occur *after* trial n (Fournier & Gallimore, 2013; Stoet & Hommel, 1999).

2 In fact, we just recently learned that in Pfister et al. (2022) a related idea was pursued albeit only reported as a side note in the Appendix. In a nutshell, they speculated that distractor offset should modulate binding effects as we do here. Yet, they did not find an effect of action-triggered distractor offset.

3 We are aware of the problem that JASP can produce somewhat varying BFs with the same constant data-set (Pfister, 2021), and hence re-ran the analyses several times with the latest version of JASP. However, the results were essentially the same in all runs.
